# Assocation between trapezium size and failure of total trapeziometacarpal prosthesis. A survival analysis

**DOI:** 10.1007/s00402-024-05525-w

**Published:** 2024-09-13

**Authors:** Clarisa Simón-Pérez, Miguel Angel Martín-Ferrero, Pedro Hernandez-Cortes, Begoña Coco Martin, Roberto S. Rosales

**Affiliations:** 1 Discipline of Orthopaedics, University of Valladolid, Valladolid, Spain; 2 Unit for Hand and Microsurgery, GECOT, La Laguna, Tenerife, Spain; 3 Upper Limb Surgery Unit, Orthopedic Surgery Department, University Hospital of Granada, Granada, Spain; 4 University of Valladolid, Valladolid, Spain; 5 Hospital Clínico Universitario, Avenida Ramón y Cajal, s/n, Valladolid, 47005 Spain

**Keywords:** Survival analysis, Total trapezium metacarpal prosthesis, Cox regression, Kaplan-Meier osteoarthritis, Trapezium bone

## Abstract

**Aims:**

To assess the survival function of cementless total trapezium metacarpal prostheses (TTMPs) at 20 years, to compare survival functions by trapezium size, and to evaluate the association between the instantaneous risk of TTMP failure and small trapezium size using a multivariate Cox regression model.

**Methods:**

This observational cohort study included 221 consecutive patients with a mean follow-up after TTMP of 137.3 months (maximum of 246 months). Kaplan-Meier and actuarial life-table methods were used to evaluate the survival function of thecohort. Kaplan-Meier survival curves were compared by trapezium size. Multivariate Cox regression analysis was used to determine the effect of potential confounders on the association between small trapezium and the instantaneous risk of TTMP failure.

**Results:**

At the end of follow-up, there was a 89.01% chance of the TTMP surviving for 246 months or more. There was an association between TTMP survival time and trapezium size showing a significant trend such that the survival curves weresignificantly higher with larger trapezium size (Mantel-Cox test, p = 0.0001; WilcoxonBreslow test, p = 0.0002; Tarone-Ware test, p = 0.0001).The unadjusted Cox regression model showed a significant association between small trapezium size (smaller than 9 mm) and the instantaneous risk of TTPM failure (HR: 7.37, 95% CI: 2.46-22.07). In the multivariate Cox analysis, “age”, “trapezium morphology”, and “complications” were confounders in the association between small trapezium size and the hazard ratio of prosthetic failure (HR = 3.76; 95% CI 0.96 to 13.82).

**Conclusion:**

These results confirm the long-term functional survival of TTMP prostheses and reveal a significant increase in trend of the survival curve with larger trapezium size. Patient age, trapezium morphology, and the presence of post-surgical complications are confounders in the association between small trapezium size and the hazard ratio of TTMP failure.

**Supplementary Information:**

The online version contains supplementary material available at 10.1007/s00402-024-05525-w.

## Introduction

Trapeziometacarpal prostheses have become an alternative surgical option to trapeziectomy or trapeziectomy with ligament reconstruction and tendon interposition, the most frequent treatments of primary thumb carpometacarpal osteoarthritis (TCMC OA) [1, 2]. They can be grouped into two main design types: joint spacer implants or partial prostheses and total trapeziometacarpal prostheses (TTMPs). These prostheses offer various advantages over other surgical approaches, including a greater reduction in pain, a faster recovery, the preservation of mobility and grip strength, and avoidance of the first metacarpal shortening produced by trapeziectomy and its variants [3–6]. However, numerous complications have been documented, sometimes early, especially when joint spacer implants or partial prostheses are utilized [2, 7].

TTMPs have demonstrated enduring clinical and functional benefits and high long-term survival rates [8–11]. However, good long-term outcomes require the correct selection of patients and implants for this technically demanding surgery [8–10], and the same technique is not suitable for all patients with TCMC OA. Their selection for TTMP is primarily related to the radiological Eaton-Littler stage [12], the presence of trapeziometacarpal arthrosis, involvement of the scaphoid-trapezium-trapezoid (STT) joint, and the age and activity of patients [8, 10, 14]. Identification of the variables that influence the long-term survival of these prostheses would enable a more precise selection of patients. It was hypothesized that trapezium size is a relevant factor in implant survival.

The objectives of this study were to assess the survival function of cementless unconstrained ball-and-socket TTMPs (Arpe^®^) up to 20 years, to compare survival functions by trapezium size, and to evaluate the association between instantaneous TTMP failure risk and small trapezium size by multivariate Cox regression analysis adjusted for confounders.

## Methods

### Study design

An observational cohort study was performed in consecutive patients undergoing cementless unconstrained ball-and-socket TTMP (Arpe^®^) surgery at our center between July 1, 1999, and August 15, 2008, who were assessed preoperatively and postoperatively at 3, 6, and 12 months and then annually.

Strengthening the Reporting of Observational Studies in Epidemiology (STROBE) guidelines [15] were followed.

### Setting & eligibility criteria

The study was approved by the ethics committee of the hospital and the ethics portal for biomedical research in Valladolid, Spain (PI 17–659).

Inclusion criteria were the presence of type III or IV TCMC OA (Eaton-Littler classification) [12] with only mild or moderate radiographic involvement of the STT joint and no pain on clinical examination (Crosby stages I and II) [13], good bone quality, and the signing of informed consent to study participation [10]. Exclusion criteria were the presence of rheumatoid or other inflammatory arthritis, symptomatic and radiographically severe OA at the STT joint (Crosby stage III) [13] or fixed hyperextension of the first metacarpophalangeal joint, occupation making high physical demands on hands (e.g., drilling or hammering), and refusal or withdrawal of consent to participation [10].

### Variables and data sources

#### Dependent variable

“Failure” of the TTMP was defined by one major criterion or two minor criteria. Major criteria were: component dislocation or loosening; visual analog scale (VAS) pain score greater than 5; and Disability of the Arm, Shoulder and Hand (DASH) questionnaire score greater than 40, using the Spanish version (Rosales et al. 2002, 2009) approved by the Institute for Work and Health in Ontario, Canada (https://dash.iwh.on.ca/available-translations). Minor criteria were partial cup loosening, with no positional changes over time; malposition of components with subluxation; and VAS pain score of 3–4 or DASH score of 30–40.

#### Independent variables

**Fig. 1:**
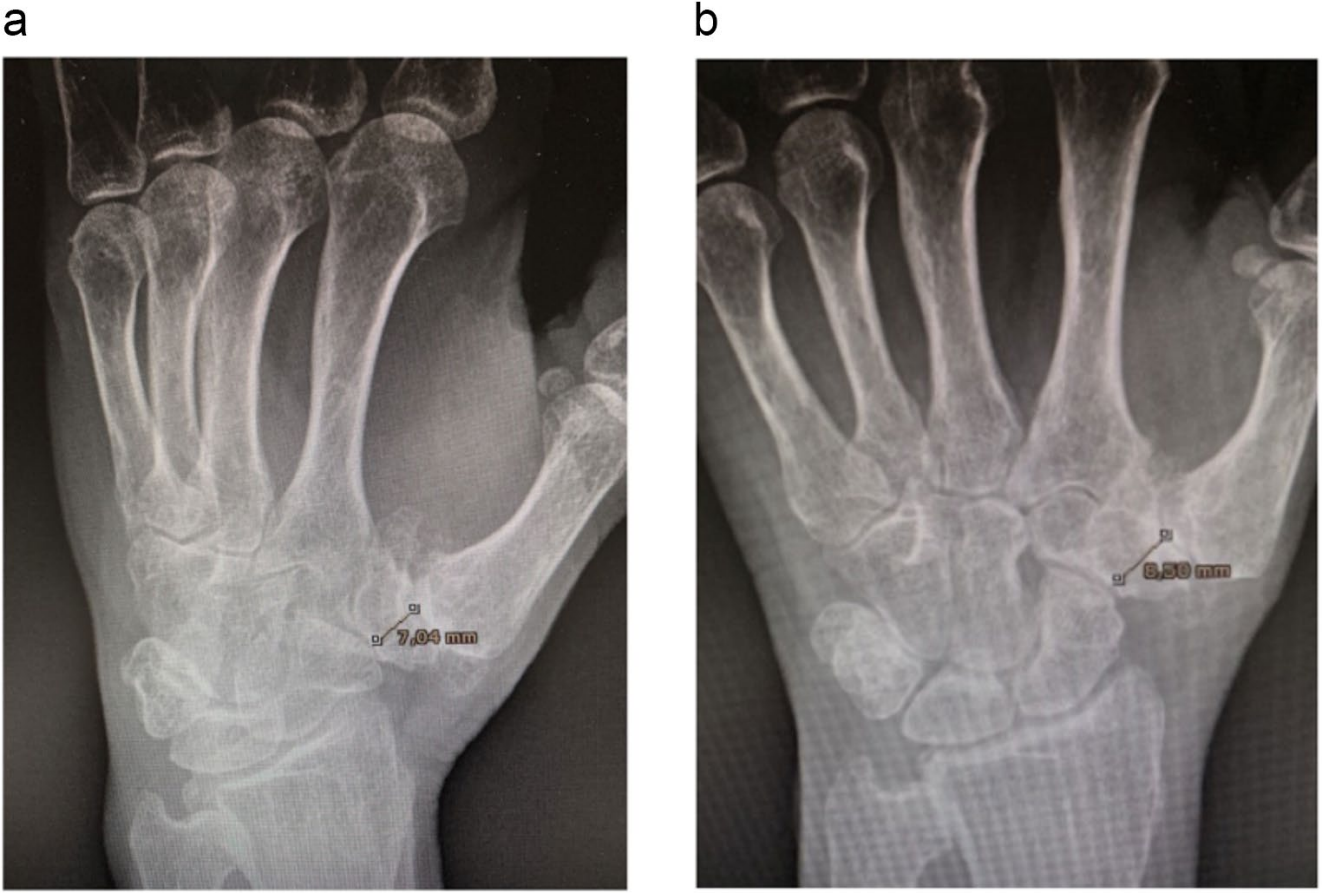
Measurement of trapezium size on the preoperative radiogram

The exposure variable was trapezium size, measured on the preoperative radiogram using a program that scales according to the distance of the radiation and classified as small (< 9 mm in both PA and oblique hand views), normal (9–12 mm in both views), or large (> 12 mm in both views) (Fig. 1). The trapezial component of the prosthesis is 9 mm wide and 4.5 mm deep.

The remaining independent variables (potential confounders) were age, gender, trapezium morphology (Trapezial dysplasia: trapezial inclination > 15º) [15], stage IV (Eaton-Littler classification) [12] with incipient STT joint involvement [13], and the presence of complications.

### Statistical analysis

Kaplan-Meier [17] and actuarial life-table methods were used to determine the survival function of the whole cohort and according to the trapezium size. A competing risk model was not developed in the absence of other causes of prosthesis failure (e.g., secondary to trauma or infection, etc.) [18]. Kaplan-Meier survival curves were compared by trapezium size using the Mantel-Cox (M-C) log rank test [19], Wilcoxon- Breslow (W-B) test [20], and Tarone-Ware (T-W) test [21], following the chi-squared distribution and establishing as null hypothesis the equality of survivor functions and absence of trend in the survivor function, setting statistical significance at *p* < 0.05.

#### Quality of follow-up

The distribution of the frequencies of the different states (Alive, Failure, or Lost) was determined at the end of the follow up period, calculating the follow-up periods for each state and expressing their minimum, maximum, mean, median, and 25th and 75th percentiles.

Multivariate Cox regression analysis for effect measurement.

1.- Selection of potential confounders. Independent variables with a p-value ≤ 0.20 in univariate Cox regression analysis were considered as confounders [22]. “Age” and “Gender” (both with *p* > 0.20) were also entered in the model due to their theoretical importance (supplementary material additional file). Consequently, “small trapezium” was considered as exposure variable and “Age”, “Gender”, “Trapezium morphology” and “Complications” as potential confounders.
2.Maximum Model (MMax). Construction of the MMax was based on the confounders and their interaction with the exposure variable (small trapezium). A Chunk test was used to assess interactions by comparing between the MMax model and the model without interactions (MMaxNoInteract), based on the likelihood ratio statistic and following the chi-squared distribution with a degree of freedom (df) equal to k (number of IVs) minus the number of interactions, establishing a level of significance of 0.05 (complete data in supplementary file). When the global Chunk test result was significant, each interaction was independently analyzed, entering those found to be statistically significant in the individual chunk test into the final “reference” model.
3.- Assessment of confounders.

Following the recommendations of Maldonado and Greenland [22], the assessment of confounders was not based on a statistical test but rather on a change of less than 10% in their effect on the hazard ratio (HR) compared with the reference model. A sensitivity analysis was conducted by comparing all reduced models to the reference model using the Stata user-command “confound” [23, 24] developed for modeling confounding in linear, logistic and Cox regression multivariate analyses (complete data in additional file). The selected adjusted model was analyzed by Cox regression analysis and compared with the unadjusted model.

4.- Diagnosis of the model.

Two assumptions are required in the Cox proportional hazard model: the proportionality assumption and a log-linear relationship.

The proportionality assumption assumes that the effect of the predictors on the HR was constant throughout the follow-up period. To verify this assumption, the interaction of each of the predictors with the survival time variable was added to the adjusted Cox model, with the null hypothesis that the coefficients of interaction between predictors (SmallTrapez Age TrapzShape Complicat) and survival time would be statistically equal to zero. The proportionality assumption was also verified by using a chi-square test to evaluate the relationship between Schoenfeld residuals and survival time, assuming proportionality when the p-value was > 0.05 [25].

According to the log-linear assumption of the Cox model, the relationship between the instantaneous incidence rate and explanatory variables must be log-linear. This was tested by using the squared linear predictor, assuming a log-linear relationship when the coefficient was not statistically significant (*p* > 0.05).

## Results

**Fig. 2:**
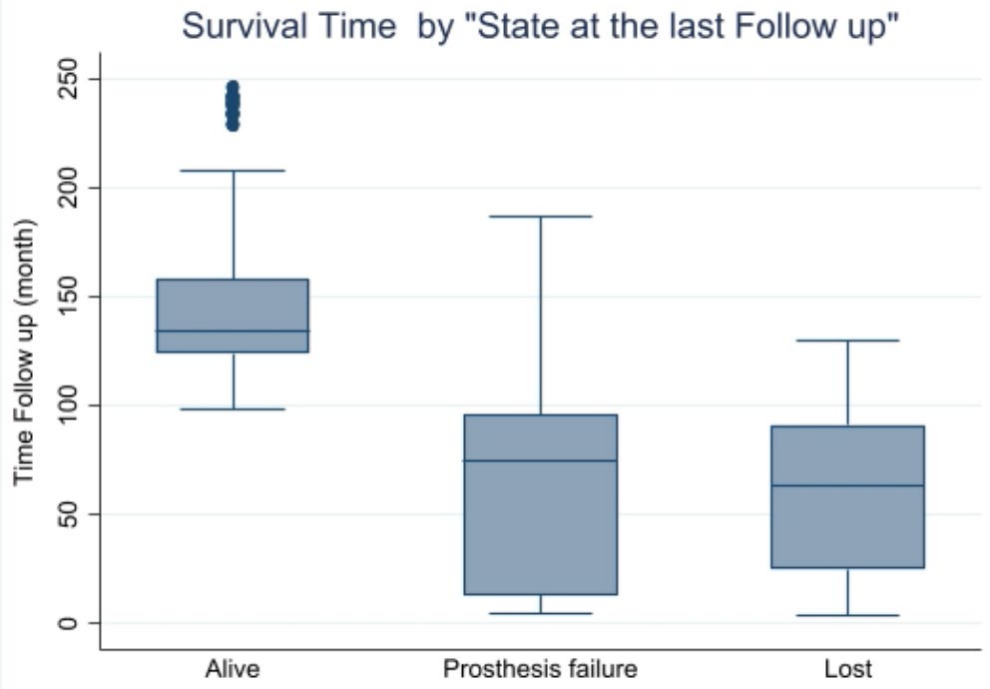
Survival time by state at the last follow up.Distribution of the frequencies of the different states (Alive, Dead,Lost) at the end of the follow-up

A total of 221 patients were consecutively enrolled in the study, 11 males (5%) and 210 females (95%) with a mean age of 58.7 years (range: 41 to 77 years). Only 14 (6.3%) patients were lost to the follow-up. The mean follow-up period was 137.3 months (range 4 to 246 months), and the maximum was 246 months. A total of 192 prostheses were still functioning at the end of the follow-up, and prosthetic failure was observed in only 15 patients (6.8%); 75% of prosthetic failures and patient losses occurred during the first 100 months (Fig. 2). Table 1 lists the minimums, maximums, means, medians, and 25th and 75th percentiles for survival times by state (alive, failure, or lost) at the end of the follow-up.

**Table 1:** Survival time (month) by last follow-up state

	*N*	Mean	Min	Max	Median	p25	p75
Alive	192	148.59	98	246	134.5	124	158
Lost	14	62.79	4	130	63	25	91
Prosthesis failure	15	61.80	5	181	72	10	84
Total	221	137.26	4	246	132	122	155

The follow-up period for the different states at the last observation in the cohort. Minimums (min), maximums (Max), means, medians, and 25th and 75th percentiles are exhibited for each state at the end of the follow-up

Fifteen patients had complications requiring removal of the prosthesis and a trapeziectomy (or variant): trapezium cup loosening in 9 patients and prosthesis dislocation in 6.

**Fig. 3:**
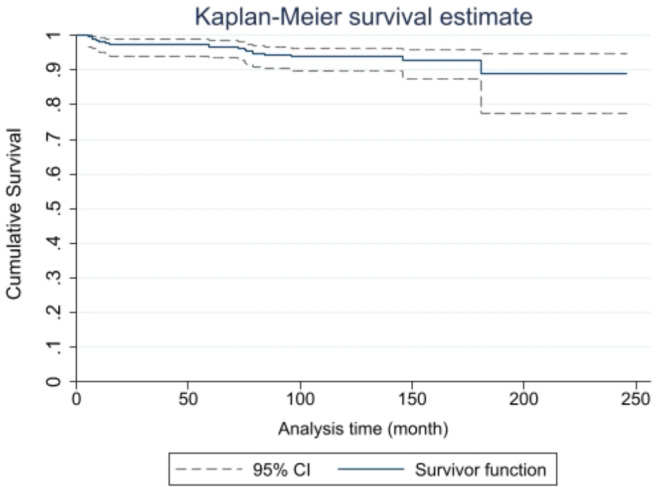
Kaplan- Meier survival estimateThe survival function of the whole cohort by the Kaplan-Meier method

According to the Kaplan-Meier survival analysis, the cumulative probability of TTMP survival was 0.968 at 64 months, 0.9396 at 120 months, and 0.8901 at 240 months (Fig. 3) (Table 2), i.e., the chance of survival was 96.8% for the first 64 months, 93.96% for the first 120 months (10 years), and 89.01% for the first 246 months (> 20 years) (complete data in supplementary material). The mean survival time (230.31 months) is a biased measure that cannot be accepted because the last observation was a censored time (alive), and it is not possible to calculate the median survival time because the cumulative available survival at the last observation was greater than 50%.

**Table 2:** Survivor function of entire cohort. Kaplan-Meier method

Time	At Risk	Fail	Lost	Survivor Function	Std. Error	[95% Conf. Int.]	
4	221	0	1	1.0000			
5	220	1	0	0.9955	0.0045	0.9682	0.9994
12	216	0	1	0.9818	0.0090	0.9523	0.9931
50	211	0	1	0.9727	0.0110	0.9402	0.9876
64	206	0	1	0.9680	0.0119	0.9341	0.9846
91	199	0	1	0.9443	0.0156	0.9040	0.9680
120	193	0	14	0.9396	0.0163	0.8982	0.9645
146	76	1	0	0.9272	0.0202	0.8755	0.9579
185	24	0	2	0.8901	0.0412	0.7759	0.9480
246	1	0	1	0.8901	0.0412	0.7759	0.9480

Survivor function = cumulative survival probability; Std = standard; [95% Conf. Int.] = 95% confident interval

**Fig. 4:**
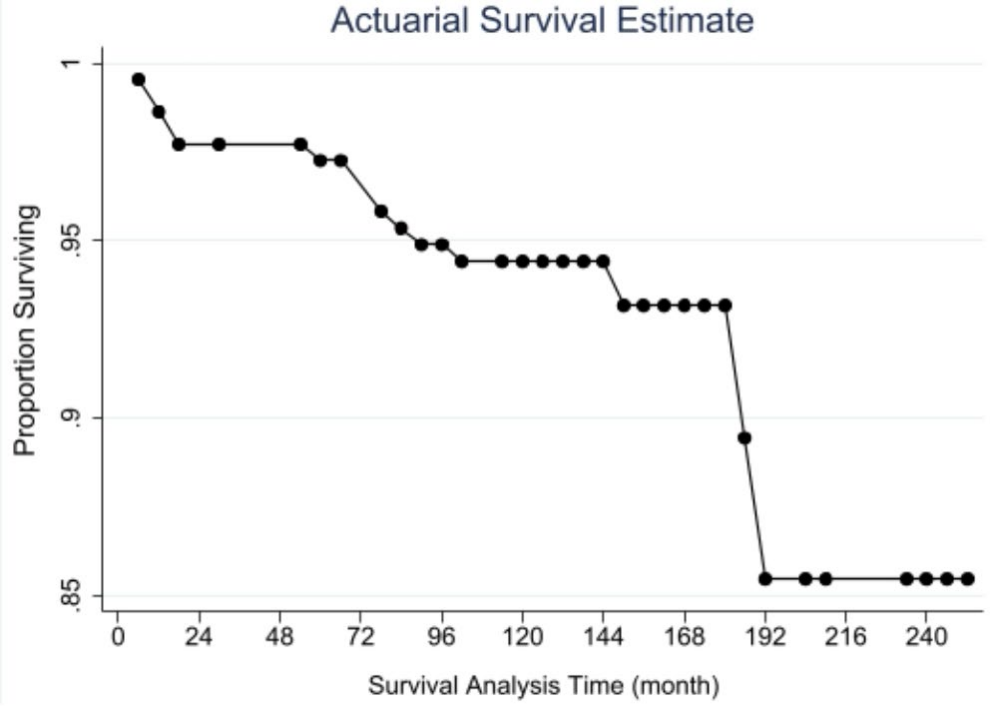
Life-table actuarial method survival. The survival function of the whole cohort by the actuarial life-tablemethod

**Fig. 5:**
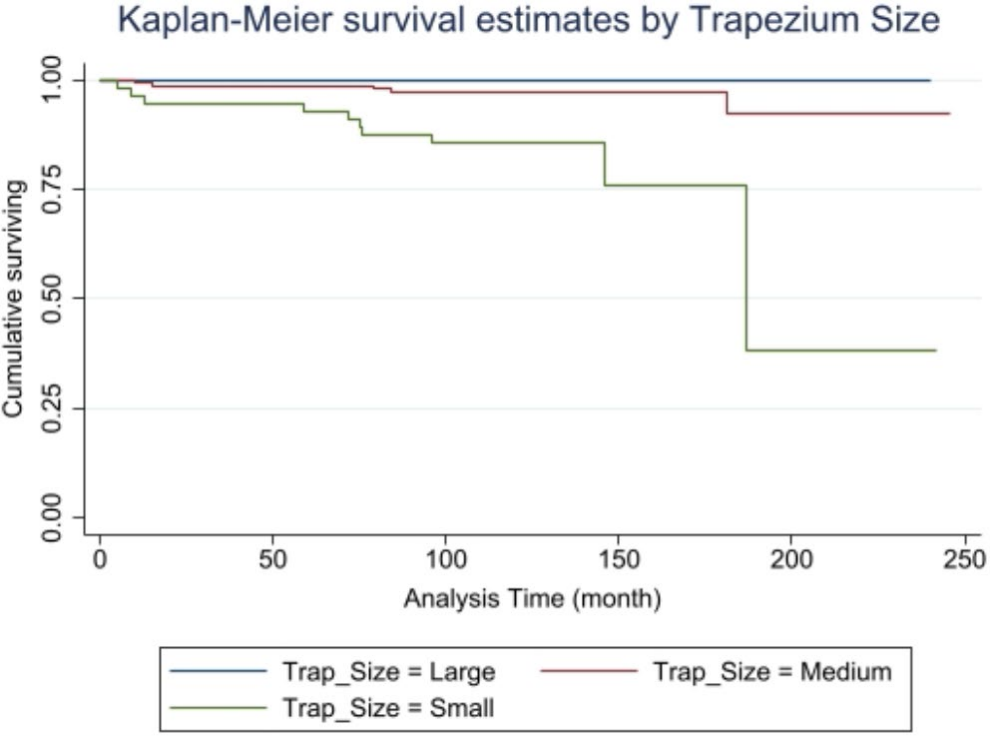
Kaplan-Meier estimates by trapezium size. Comparison of Kaplan-Meier survival curves by trapezium size(Large, Medium, Small)

Figure 4 and 5; Table 3 exhibit the cumulative TTMP survival probability calculated by the actuarial life-table method, showing that the chance of survival was 93.96% up to 120 months (10 years) and 88.84% up to 252 months (> 20 years).

**Table 3:** Survival function. The actuarial method. Whole cohort

		Initial			Std.		[95% Conf. Int.]	
Interval		Total	Deaths	Lost	Survival	Error		
0	6	221	1	1	0.9955	0.0045	0.9682	0.9994
6	12	219	3	0	0.9818	0.0090	0.9523	0.9931
12	18	216	2	2	0.9727	0.0110	0.9402	0.9876
54	60	210	1	0	0.9681	0.0119	0.9342	0.9846
90	96	199	0	1	0.9444	0.0156	0.9041	0.9680
138	144	91	0	12	0.9396	0.0163	0.8982	0.9645
174	180	31	0	6	0.9271	0.0203	0.8751	0.9579
246	252	1	0	1	0.8884	0.0425	0.7699	0.9479

As depicted in Fig. 4, TTMP survival curves were higher with larger trapezium size and the trend was significant (M-test, *p* = 0.0001; W-B test, *p* = 0.0002; T-W test, *p* = 0.0001) (Supplementary material). In the unadjusted Cox regression model, small trapezium size was significantly associated with the instantaneous risk of TTMP failure (HR: 7.37; 95% CI: 2.46 to 22.07) (Table 4). In the multivariate analysis, the association between the instantaneous risk of prosthetic failure and small trapezium size was best explained by the model adjusted for “age”, “trapezium morphology” and “complications”. When results were adjusted for these confounding variables, the HR of prosthetic failure was 3.64-fold higher when the trapezium was small, although the difference was only close to statistical significance (95% CI: 0.96 to 13.82) (*p* = 0.058) (Table 4). Diagnosis of the model confirmed that the proportional hazard assumption was met, based on Schoenfeld residuals (*p* = 0.9788) and on the interaction between the survival time and independent variables (*p* = 0.8743), and that the log-linear assumption was also fulfilled (*p* = 0.815) (complete data in Additional file Supplementary material).

**Table 4:** Association between “Small Trapezium” and the hazard function of the TTMP

A.- Unadjusted Model						
_t	Hazard Ratio	Std. Err.	z	P>|z|	[95% Conf. Interval]	
SmallTrapez	7.373491	4.123877	3.57	0.000	2.463824	22.06667
*B.- Adjusted Model*						
_t	Hazard Ratio	Std. Err.	z	P>|z|	[95% Conf. Interval]	
SmallTrapez	3.639171	2.477723	1.90	0.058	0.9582161	13.82107
Age	0.9520679	0.0369557	-1.27	0.206	0.8823228	1.027326
TrapzMorph	0.8220775	0.3280789	-0.49	0.623	0.37602	1.797275
Complicat	7.99e + 16	1.52e + 24	0.00	1.000	0	.

SmallTrapez = small trapezium; TrapMorph = trapezium morphology; Complicat = post-surgical complications

## Discussion

This 20-year follow-up study of more than 200 cementless unconstrained ball-and-socket TTMPs (Arpe^®^) confirms their long-term functional durability, observing an 89.01% chance of their survival for 246 months or longer. Another clinically relevant finding was that TTMP survival curves were significantly higher with larger trapezium size. Among 15 multivariate Cox regression models considered in the confounder analysis, the association between the instantaneous incidence rate of prosthetic failure and small trapezium was best explained by the model adjusted for age, trapezium morphology, and the presence of complications.

None of the multiple surgical techniques available to treat TCMC OA have proven superior to any other [2]. However, questions have been raised about the suitability of TTMP for all patients with TMTC OA, given that a proper surgical indication is a crucial factor influencing the outcome [15]. The ideal candidate for TTMP is a patient with Eaton and Littler stage III TMTC OA [12], no involvement of neighboring joints, trapezium size of 9 mm or larger, and good bone quality who does not engage in physically demanding work [8–10].

In the present study, the cementless unconstrained ball-and-socket TTMP (Arpe^®^) survival rate was 93.9% at a mean follow-up of 137.3 months. Studies of patients with similar surgical indications and characteristics have reported good short-term and long-term (> 10-year) outcomes, with high survival rates [8–11, 26–29], although most had a retrospective design and heterogeneous sample [30, 31], and none conducted survival analysis using a multivariate Cox model. Among previous investigations, a retrospective clinical and radiographic study described a 12-year survival rate of 88% in a series of 191 Maia prostheses after a median follow-up of 69 months, although the follow-up period ranged widely between 17 days to 140 months [28] and a Kaplan-Meier survival probability of 96% was observed in a series of 166 Arpe prostheses, after a mean follow-up of 80 months [27]. In another study, a survival rate of 91% was reported for 64 Roseland prostheses after a mean follow-up of 12.5 years, although concerns were raised about a possible abrupt drop in survival with longer follow-up given radiographic findings of a high rate of asymptomatic periprosthetic osteolysis, which may be related to the semi-constrained design of the prosthesis [32]. In a case-control study, Kaplan-Meier survival analysis showed significantly lower implant survival in male *versus* female patients with Ivory Arthroplasty for Trapeziometacarpal Joint Arthritis [33].

There have been few long-term prospective studies of modular, cementless, non-constrained total prostheses. After a minimum follow-up of 10 years, one series of 110 Ivory prostheses had a survival rate of 95% [11] and another series of 26 Ivory prostheses a rate of 85% [31], and both studies reported the same satisfactory long-term clinical, functional, and radiological outcomes as observed in the present study [8,10]. However, the present results contrast with the observation by Druel et al. [34] of a linear reduction in implant survival rate over time (from 83% at 5 years to 50% at 30 years) in a series of 41 trapeziometacarpal prostheses of the same type (Arpe modular non-constrained single-mobility prosthesis).

Application of the actuarial life table approach revealed that 75% of prosthetic failures and patient losses occurred in the first 100 months post-surgery and that the cumulative survival probability of these uncemented and unconstrained TTMPs was 93.96% up to 120 months (10 years) and 88.84% up to 252 months (> 20 years).

Study strengths include the prospective design, large sample size, long consecutive follow-up of patients, small number of dropouts, and application of adjusted multivariate Cox models that met all necessary assumptions. It contributes the clinically relevant finding that the selection of candidates for TTMP should consider the size of the trapezium as well as its shape and the patient’s age.

Study limitations include the measurement of trapezium size on two-dimensional radiograms rather than three-dimensional CT scans, which are currently preferred. Furthermore, the TM joint is positioned anteriorly to the carpus and tilted radially; hence, a normal radiological examination of the hand provides an approximate representation because it is performed in oblique planes. In addition, the inter-observer variability was high for the Eaton-Littler classification, which was independently measured by only two researchers (CSP, MAMF); Finally, all TTMPs were performed by two experienced surgeons at a single center, limiting the external validity of these findings.

In conclusion, based on the results of this study, TTMP prostheses have a long-term functional survival. The survival curve rises with the increase in trapezium size. Patient age, trapezium morphology, and the presence of post-surgical complications are confounders in the association between small trapezium size and the hazard ratio of TTMP failure.”

## Electronic supplementary material

Below is the link to the electronic supplementary material.

Supplementary Material 1

## Data Availability

The datasets used and/or analysed during the current study available from the corresponding author on reasonable request.

## References

[1] Martou G, Veltri K, Thoma A (2004) Surgical treatment of osteoarthritis of the carpometacarpal joint of the thumb: a systematic review. Plast Reconstr Surg 114(2):421 − 32. 10.1097/01.prs.0000131989.86319.b1 15277809

[2] Vermeulen GM, Slijper H, Feitz R, Hovius SE, Moojen TM, Selles RW (2011) Surgical management of primary thumb carpometacarpal osteoarthritis: a systematic review. J Hand Surg Am 36(1):157 − 69. 10.1016/j.jhsa.2010.10.028 21193136

[3] Cebrian-Gomez R, Lizaur-Utrilla A, Sebastia-Forcada E, Lopez-Prats FA (2019) Outcomes of cementless joint prosthesis versus tendon interposition for trapeziometacarpal osteoarthritis: a prospective study. J Hand Surg Eur 44(2):151–158. 10.1177/1753193418787151 30016903

[4] Ulrich-Vinther M, Puggaard H, Lange B (2008) Prospective 1-year follow-up study comparing joint prosthesis with tendon interposition arthroplasty in treatment of trapeziometacarpal osteoarthritis. J Hand Surg Am 33(8):1369-77. 10.1016/j.jhsa.2008.04.028 18929203

[5] Jurča J, Němejc M, Havlas V (2016) Srovnání výsledků operační léčby rhizartrózy metodou interpoziční artroplastiky dle Burtona-Pellegriniho a implantací trapeziometakarpální endoprotézy [Surgical Treatment for Advanced Rhizarthrosis. Comparison of Results of the Burton-Pellegrini Technique and Trapeziometacarpal Joint Arthroplasty]. Acta Chir Orthop Traumatol Cech. 83(1):27–31 26936062

[6] Jager T, Barbary S, Dap F, Dautel G (2013) Analyse de la douleur postopératoire et des résultats fonctionnels précoces dans le traitement de la rhizarthrose. Étude prospective comparative de 74 patientes trapézectomie-interposition vs prothèse MAIA (^®^) [Evaluation of postoperative pain and early functional results in the treatment of carpometacarpal joint arthritis. Comparative prospective study of trapeziectomy vs. MAIA (^®^) prosthesis in 74 female patients]. Chir Main 32(2):55–62. 10.1016/j.main.2013.02.004 23561855

[7] Huang K, Hollevoet N, Giddins G (2015) Thumb carpometacarpal joint total arthroplasty: a systematic review. J Hand Surg Eur Vol 40(4):338 − 50. 10.1177/1753193414563243 25600851

[8] Martin-Ferrero M (2014) Ten-year long-term results of total joint arthroplasties with ARPE^®^ implant in the treatment of trapeziometacarpal osteoarthritis. J Hand Surg Eur 39(8):826 − 32. 10.1177/1753193413516244 24334554

[9] Vissers G, Goorens CK, Vanmierlo B, Bonte F, Mermuys K, Fils JF, Goubau JF (2019) Ivory arthroplasty for trapeziometacarpal osteoarthritis: 10-year follow-up. J Hand Surg Eur 44(2):138–145. 10.1177/1753193418797890 30227766

[10] Martin-Ferrero M, Simón-Pérez C, Coco-Martín MB, Vega-Castrillo A, Aguado-Hernández H, Mayo-Iscar A (2020) Trapeziometacarpal total joint arthroplasty for osteoarthritis: 199 patients with a minimum of 10 years follow-up. J Hand Surg Eur 45(5):443–451. 10.1177/1753193419871660 31495260

[11] Tchurukdichian A, Guillier D, Moris V, See LA, Macheboeuf Y (2020) Results of 110 IVORY^®^ prostheses for trapeziometacarpal osteoarthritis with a minimum follow-up of 10 years. J Hand Surg Eur 45(5):458–464. 10.1177/1753193419899843 31992116

[12] Eaton RG, Littler JW (1973) Ligament reconstruction for the painful thumb carpometacarpal joint. J Bone Joint Surg Am 55(8):1655-66. 4804988

[13] Crosby EB, Linscheid RL, Dobyns JH. (1978) Scaphotrapezial trapezoidal arthrosis. J Hand Surg Am 3(3):223 − 34. 10.1016/s0363-5023(78)80086-0 659818

[14] de la Caffinière JY (2001) Facteurs de longévité des prothèses totales trapézométacarpiennes [Longevity factors in total trapezometacarpal prostheses]. Chir Main 20(1):63 − 7. 10.1016/s1297-3203(01)00015-4 11291321

[15] Vandenbroucke JP, Elm E von, Altman DG, Gøtzsche PC, Mulrow CD, Pocock SJ, et al (2014) Strengthening the Reporting of Observational Studies in Epidemiology (STROBE): Explanation and elaboration. Int J Surg 12(12):1500–1524. 10.1016/j.ijsu.2014.07.014 25046751

[16] Van Royen K, Scheerlinck T, Van Royen A, De Keyzer PB, Baetslé A, Goubau J.(2022) Defining trapezial dysplasia - analysis of trapezial inclination in a normal population. J Hand Surg Eur 47(6):618–625. doi: 10.1177/17531934221075921 10.1177/17531934221075921 35102775

[17] Kaplan EL, Meier P (1958) Nonparametric Estimation from Incomplete Observations. J Am Stat Assoc.53(282):457–481.

[18] Gooley TA, Leisenring W, Crowley J, Storer BE (1999) Estimation of failure probabilities in the presence of competing risks: New representations of old estimators. Stat Med. 18(6):695–706. 10.1002/(sici)1097-0258(19990330)18:6<695::aid-sim60>3.0.co;2-o 10204198

[19] Mantel N (1966) Evaluation of survival data and two new rank order statistics arising in its consideration. Cancer Chemother reports 50(3):163–170. 5910392

[20] Breslow N (1970) A Generalized Kruskal-Wallis Test for Comparing K Samples Subject to Unequal Patterns of Censorship. Biometrika. 57(3):579–594.

[21] Tarone RE, Ware J (1977) On Distribution-Free Tests for Equality of Survival Distributions. Biometrika. 64(1):156–160.

[22] Maldonado G, Greenland S. (1993) Simulation study of confounder-selection strategies. Am J Epidemiol 138(11):923–936. 10.1093/oxfordjournals.aje.a116813 8256780

[23] Doménech JM, Navarro JB (2020) Find the best subset for Linear, Logistic and Cox Regression: User-written command confound for Stata [computer program]. V1.1.7. Bellaterra: Universitat Autònoma de Barcelona; http://metodo.uab.cat/stata.

[24] Rosales RS, Atroshi I (2023) Clinical research in hand surgery: handling confounding and effect modification. J Hand Surg Eur 48(7):681–686. 10.1177/17531934231151529 36864782

[25] Schoenfeld, D. (1982) Partial Residuals for the Proportional Hazards Regression Model. Biometrika, 69, 239–241. 10.1093/biomet/69.1.239

[26] Brutus JP, Kinnen L (2004) Remplacement prothétique total de la trapézométacarpienne au moyen de la prothèse ARPE dans le traitement de la rhizarthrose: notre expérience à court terme dans une série personnelle de 63 cas consécutifs [Short term results of total carpometacarpal joint replacement surgery using the ARPE implant for primary ostearthritis of the thumb]. Chir Main. 23(5):224-8. 10.1016/j.main.2004.08.001 15573875

[27] Cootjans K, Vanhaecke J, Dezillie M, Barth J, Pottel H, Stockmans F (2017) Joint Survival Analysis and Clinical Outcome of Total Joint Arthroplasties with the ARPE Implant in the Treatment of Trapeziometacarpal Osteoarthritis with a Minimal Follow-Up of 5 Years. J Hand Surg Am. 42(8):630–638. 10.1016/j.jhsa.2017.05.007 28666676

[28] Chiche L, Chammas PE, Vial D’Allais P, Lazerges C, Coulet B, Chammas M (2023) Long-term survival analysis of 191 MAÏA^®^ prostheses for trapeziometacarpal arthritis. J Hand Surg Eur 48(2):101–107. 10.1177/17531934221136442 36329561

[29] Toffoli A, Degeorge B, Cloquell Y, Teissier P, Teissier J. (2024) MAÏA Trapeziometacarpal Joint Arthroplasty: Clinical and Radiological Outcomes of 76 Patients With More Than 10 Years of Follow-Up. J Hand Surg Am. S0363-5023(24)00174-6. 10.1016/j.jhsa.2024.03.019 38935000

[30] Fauquette PJ, Deken-Delannoy V, Chantelot C, Saab M. (2023) The ISIS^®^ prosthesis in 77 cases of trapeziometacarpal arthritis: outcomes and survival at a minimum follow-up of 5 years. J Hand Surg Eur Vol.48(2):108–114. 10.1177/17531934221123166 36165407

[31] Frey PE, Bühner C, Falkner F, Harhaus L, Panzram B. (2024) Mid- and long-term clinical results of the Elektra and Moovis prosthesis for trapeziometacarpal joint replacement. BMC Musculoskelet Disord. 25(1):332. 10.1186/s12891-024-07439-5 PMC11044538 38664698

[32] Semere A, Vuillerme N, Corcella D, Forli A, Moutet F (2015) Results with the Roseland (^®^) HAC trapeziometacarpal prosthesis after more than 10 years. Chir Main.34(2):59–66. 10.1016/j.main.2015.01.004 25769771

[33] Vanmierlo B, Buitenweg J, Vanmierlo T, Van Royen K, Bonte F, Goubau J. (2022) Ivory Arthroplasty for Trapeziometacarpal Joint Arthritis in Men: Analysis of Clinical Outcome and Implant Survival. Hand (N Y). 17(3):440–446. 10.1177/1558944720930297 PMC9112725 32697106

[34] Druel T, Cievet-Bonfils M, Comtet JJ, Gazarian A. (2024) 31 years survival rate of ARPE^®^ single-mobility prosthesis in trapeziometacarpal osteoarthritis. J Hand Surg Eur Vol. 49(7):914–916. 10.1177/17531934231221692 38114074

