# Supplementary material for: Assocation between trapezium size and failure of total trapeziometacarpal prosthesis. A survival analysis
Source: Arch Orthop Trauma Surg. 2024 Sep 13;144(9):4275–82. doi: 10.1007/s00402-024-05525-w (PMC11564239; doi:10.1007/s00402-024-05525-w)
Supplement: Supplementary file 1 — Supplementary Material 1 [file 402_2024_5525_MOESM1_ESM.docx]

Supplementary material

**(R)**

**/ /**  **/ /**  **/**

**/ / / / / / / 16.1 Copyright 1985-2019 StataCorp LLC Statistics/Data analysis StataCorp**

**4905 Lakeway Drive**

**College Station, Texas 77845 USA**

**800-STATA-PC https://**[**www.stata.com**](http://www.stata.com/) **979-696-4600** [**stata@stata.com**](mailto:stata@stata.com)

**979-696-4601 (fax)**

**Stata license: Single-user perpetual Serial number: 301606250749**

**Licensed to: Roberto S Rosales**

**Unit for Hand and Micro Surgery**

**Notes:**

**. describe**

**Contains data from C:\Users\Usuario\AppData\Local\Temp\ST_4d4_000001.tmp**

| **obs:** | **221** | |  | | |
| --- | --- | --- | --- | --- | --- |
| **vars:** | **26** | | **6 Sep 2023 14:46** | | |
|  | **storage** | | **display** | **value** |  |
| **variable name** | **type** | | **format** | **label** | **variable label** |
| **ID** | | **int** | **%10.0g** |  | **ID** |
| **Number_Hist** | | **str9** | **%9s** |  | **Number_Hist** |
| **FNac** | | **int** | **%td..** |  | **Date of born** |
| **FCirg** | | **int** | **%td..** |  | **Surgery date** |
| **Ffin** | | **int** | **%td..** |  | **Date of last follow-up** |
| **EstadFin** | | **byte** | **%23.0g** | **dEstadFin** |  |
|  | |  |  |  | **Last Follow up state** |
| **Age** | | **float** | **%9.0g** |  | **Age (decimal years)** |
| **Gender** | | **byte** | **%10.0g** | **dGender** | **Gender** |
| **Trap_Size** | | **byte** | **%10.0g** | **dTrap_Size** |  |
|  | |  |  |  | **Trap_Size** |
| **TrapzShape** | | **byte** | **%21.0g** | **dTrapzShape** |  |
|  | |  |  |  | **Trapezium Shape** |
| **Eaton_grade** | | **byte** | **%9.0g** | **dEaton** | **Grade IV Eaton** |
| **PainEVA** | | **byte** | **%10.0g** |  | **Pain EVA** |
| **DASH** | | **str17** | **%17s** |  | **DASH** |
| **Kapndji** | | **byte** | **%10.0g** |  | **Kapndji** |
| **radialabd** | | **byte** | **%10.0g** |  | **radial abd** |
| **_NMiss** | | **float** | **%9.0g** |  | **Total missing values by subject** |
| **TSm** | | **float** | **%9.0g** |  | **Time Follow up (month)** |
| **_st** | | **byte** | **%8.0g** |  | **1 if record is to be used; 0 otherwise** |
| **_d** | | **byte** | **%8.0g** |  | **1 if failure; 0 if censored** |
| **_t** | | **int** | **%10.0g** |  | **Analysis time when record ends** |
| **_t0** | | **byte** | **%10.0g** |  | **Analysis time when record begins** |
| **SmallTrapez** | | **byte** | **%9.0g** | **dNoYes** | **Samall trapezium** |
| **GradeIV_Eaton** | | **byte** | **%9.0g** |  | **RECODE of Eaton_grade (Grades Eaton)** |
| **Complicat** | | **byte** | **%9.0g** | **dNoYes** | **Complication** |
| **_est_MMax** | | **byte** | **%8.0g** |  | **esample() from estimates store** |
| **_est_MMaxNoIn~c** | | **byte** | **%8.0g** |  | **esample() from estimates store** |

**Sorted by: Trap_Size**

**Note: Dataset has changed since last saved.**

**. statmis**

**STATISTICS OF MISSING VALUES**

| **Variable** | **Missing values System User Percent** | | | **Not Appl.** | **Valid Freq.** | **values Percent** | **Mean** | **Min** | **Max** |
| --- | --- | --- | --- | --- | --- | --- | --- | --- | --- |
| **ID** | **0** | **0** | **0.00** | **0** | **221** | **100.00** | **111** | **1** | **221** |
| **Number_Hist** | **0** |  | **0.00** |  | **221** | **100.00** |  |  |  |
| **age** | **0** | **0** | **0.00** | **0** | **221** | **100.00** | **58.72398** | **41** | **77** |
| **FNac** | **0** | **0** | **0.00** | **0** | **221** | **100.00** |  | **01/12/1924** | **16/03/1965** |
| **FCirg** | **0** | **0** | **0.00** | **0** | **221** | **100.00** |  | **08/07/1999** | **13/08/2008** |
| **Ffin** | **0** | **0** | **0.00** | **0** | **221** | **100.00** |  | **12/05/2000** | **26/05/2021** |
| **EstadFin** | **0** | **0** | **0.00** | **0** | **221** | **100.00** | **.2579186** | **0** | **3** |
| **Gender** | **0** | **0** | **0.00** | **0** | **221** | **100.00** | **.0497738** | **0** | **1** |
| **Eaton** | **0** | **0** | **0.00** | **0** | **221** | **100.00** | **3.108597** | **3** | **4** |
| **Trap_Size** | **0** | **0** | **0.00** | **0** | **221** | **100.00** | **1.221719** | **0** | **2** |
| **DolorEVA** | **0** | **0** | **0.00** | **0** | **221** | **100.00** | **1.235294** | **0** | **6** |
| **DASH** | **0** |  | **0.00** |  | **221** | **100.00** |  |  |  |
| **Kapndji** | **0** | **0** | **0.00** | **0** | **221** | **100.00** | **9.656109** | **7** | **10** |
| **radialabd** | **0** | **0** | **0.00** | **0** | **221** | **100.00** | **46.31222** | **20** | **60** |
| **complicaci~s** | **0** | **0** | **0.00** | **0** | **221** | **100.00** | **1.208145** | **1** | **5** |
| **TrapzShape** | **0** | **0** | **0.00** | **0** | **221** | **100.00** | **.4117647** | **0** | **2** |
| **Eaton_grade** | **0** | **0** | **0.00** | **0** | **221** | **100.00** | **2.108597** | **2** | **3** |
| **Total** | **0** | **0** | **0.00** | **0** | **3757** | **100.00** |  |  |  |

**Total missing values by subject**

**_NMiss Freq. Percent**

**0**

**221 100.00**

**Total**

**221 100.00**

**A new variable named _NMiss has been added to the dataset.**

**It can be used to identify and drop observations with missing values**

Quality of Follow –up

### . tab EstadFin

| **Last Follow up state** | **Freq.** | **Percent** | **Cum.** |
| --- | --- | --- | --- |
| **Alive** | **192** | **86.88** | **86.88** |
| **Prosthesis failure** | **15** | **6.79** | **93.67** |
| **Lost** | **14** | **6.33** | **100.00** |
| **Total** | **221** | **100.00** |  |

A total of 6.8% (15/221) of prosthesis failures were observed, a total loss or abandonment of 6.3% (very low) and a total of 192 prostheses were still working (alive) at the end of follow-up, which was a maximum of 246 months with a mean follow-up of 138.1 (SD 45.3) months. (See tables below).

**. tabstat TSm, statistics(count min max mean median p25 p75 ) by (EstadFin)**

**Summary for variables: TSm**

**by categories of: EstadFin (Last Follow up state)**

| **EstadFin** | **N** | **min** | **max** |  | **mean** | **p50** | **p25** | **p75** |
| --- | --- | --- | --- | --- | --- | --- | --- | --- |
| **Alive** | **192** | **98** | **246** |  | **148.5885** | **134.5** | **124** | **158** |
| **Prosthesis failu** | **15** | **5** | **181** |  | **61.8** | **72** | **10** | **84** |
| **Lost** | **14** | **4** | **130** |  | **62.78571** | **63** | **25** | **91** |
|  |  |  |  |  |  |  |  |  |
| **Total** | **221** | **4** | **246** |  | **137.2624** | **132** | **122** | **155** |
|  |  |  |  |  |  |  |  |  |

**. sum TSm**

| **Variable** | **Obs** | **Mean** | **Std. Dev.** | **Min** | **Max** |
| --- | --- | --- | --- | --- | --- |
| **TSm** | **221** | **137.2624** | **46.02799** | **4** | **246** |


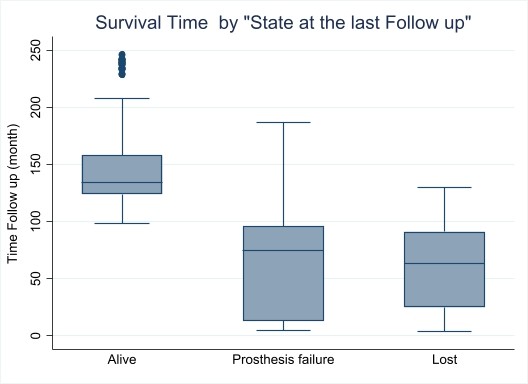


## Definition of survival data

**. stset TSm, failure(EstadFin==1)**

**failure event: EstadFin == 1 obs. time interval: (0, TSm] exit on or before: failure**

| **221** | **total observations** |  | |
| --- | --- | --- | --- |
| **0** | **Exclusions** |  |  |
| **221** | **observations remaining, representing** |  |  |
| **15** | **failures in single-record/single-failure data** |  |  |
| **30,335** | **total analysis time at risk and under observation**  **at risk from t** | **=** | **0** |
|  | **earliest observed entry t** | **=** | **0** |
|  | **last observed exit t** | **=** | **246** |

## Completed Cumulative Survival Probabiliies. Kaplan-Meier method.

**Entire Cohort**

The cumulative probability of survival of the Arpe prosthesis at 64 months was 0.968, which is interpreted as there being a 96.8% chance that an Arpe prosthesis will take more than 64 months to fail. In other words, there is a 96.8% chance that the Arpe prosthesis will survive the first 64 months, 93.96% the first 120 months (10 years), and 89.01% the first 246 months (>20 years). The mean survival (230.31 month) should not be accepted since it is a biased measure since the last observation was a censored time (alive). The median cannot be calculated either since at the end of the last observation the cumulative available survival was greater than 50%.

**. sts list, survival**

| **failure**  **analysis time** | | **_d:**  **_t:** | **EstadFin**  **TSm** | **== 1** | | |
| --- | --- | --- | --- | --- | --- | --- |
| **Time** | **At Risk** | **Fail** | **Lost** | **Survivor Function** | **Std. Error** | **[95% Conf. Int.]** |

| **4** |  |  | **221** |  | **0** | **1** |  |  | **1.0000** |  | **.** |  | **.** |  | **.** |
| --- | --- | --- | --- | --- | --- | --- | --- | --- | --- | --- | --- | --- | --- | --- | --- |
| **5** |  |  | **220** |  | **1** | **0** |  |  | **0.9955** |  | **0.0045** |  | **0.9682** |  | **0.9994** |
| **7** |  |  | **219** |  | **1** | **0** |  |  | **0.9909** |  | **0.0064** |  | **0.9641** |  | **0.9977** |
| **9** |  |  | **218** |  | **1** | **0** |  |  | **0.9864** |  | **0.0078** |  | **0.9583** |  | **0.9956** |
| **10** |  |  | **217** |  | **1** | **0** |  |  | **0.9818** |  | **0.0090** |  | **0.9523** |  | **0.9931** |
| **12** |  |  | **216** |  | **0** | **1** |  |  | **0.9818** |  | **0.0090** |  | **0.9523** |  | **0.9931** |
| **13** |  |  | **215** |  | **1** | **1** |  |  | **0.9773** |  | **0.0101** |  | **0.9462** |  | **0.9905** |
| **15** |  |  | **213** |  | **1** | **0** |  |  | **0.9727** |  | **0.0110** |  | **0.9402** |  | **0.9876** |
| **25** |  |  | **212** |  | **0** | **1** |  |  | **0.9727** |  | **0.0110** |  | **0.9402** |  | **0.9876** |
| **50** |  |  | **211** |  | **0** | **1** |  |  | **0.9727** |  | **0.0110** |  | **0.9402** |  | **0.9876** |
| **59** |  |  | **210** |  | **1** | **0** |  |  | **0.9680** |  | **0.0119** |  | **0.9341** |  | **0.9846** |
| **62** |  |  | **209** |  | **0** | **1** |  |  | **0.9680** |  | **0.0119** |  | **0.9341** |  | **0.9846** |
| **63** |  |  | **208** |  | **0** | **2** |  |  | **0.9680** |  | **0.0119** |  | **0.9341** |  | **0.9846** |
| **64** |  |  | **206** |  | **0** | **1** |  |  | **0.9680** |  | **0.0119** |  | **0.9341** |  | **0.9846** |
| **72** |  |  | **205** |  | **1** | **0** |  |  | **0.9633** |  | **0.0127** |  | **0.9280** |  | **0.9815** |
| **74** |  |  | **204** |  | **0** | **1** |  |  | **0.9633** |  | **0.0127** |  | **0.9280** |  | **0.9815** |
| **75** |  |  | **203** |  | **1** | **0** |  |  | **0.9586** |  | **0.0135** |  | **0.9219** |  | **0.9782** |
| **76** |  |  | **202** |  | **1** | **0** |  |  | **0.9538** |  | **0.0143** |  | **0.9159** |  | **0.9749** |
| **79** |  |  | **201** |  | **1** | **0** |  |  | **0.9491** |  | **0.0150** |  | **0.9099** |  | **0.9715** |
| **84** |  |  | **200** |  | **1** | **0** |  |  | **0.9443** |  | **0.0156** |  | **0.9040** |  | **0.9680** |
| **91** |  |  | **199** |  | **0** | **1** |  |  | **0.9443** |  | **0.0156** |  | **0.9040** |  | **0.9680** |
| **96** |  |  | **198** |  | **1** | **0** |  |  | **0.9396** |  | **0.0163** |  | **0.8982** |  | **0.9645** |
| **98** |  |  | **197** |  | **0** | **1** |  |  | **0.9396** |  | **0.0163** |  | **0.8982** |  | **0.9645** |
| **108** |  |  | **196** |  | **0** | **1** |  |  | **0.9396** |  | **0.0163** |  | **0.8982** |  | **0.9645** |
| **118** |  |  | **195** |  | **0** | **1** |  |  | **0.9396** |  | **0.0163** |  | **0.8982** |  | **0.9645** |
| **119** |  |  | **194** |  | **0** | **1** |  |  | **0.9396** |  | **0.0163** |  | **0.8982** |  | **0.9645** |
| **120** |  |  | **193** |  | **0** | **14** |  |  | **0.9396** |  | **0.0163** |  | **0.8982** |  | **0.9645** |
| **121** |  |  | **179** |  | **0** | **11** |  |  | **0.9396** |  | **0.0163** |  | **0.8982** |  | **0.9645** |
| **122** |  |  | **168** |  | **0** | **4** |  |  | **0.9396** |  | **0.0163** |  | **0.8982** |  | **0.9645** |
| **123** |  |  | **164** |  | **0** | **11** |  |  | **0.9396** |  | **0.0163** |  | **0.8982** |  | **0.9645** |
| **124** |  |  | **153** |  | **0** | **9** |  |  | **0.9396** |  | **0.0163** |  | **0.8982** |  | **0.9645** |
| **125** |  |  | **144** |  | **0** | **2** |  |  | **0.9396** |  | **0.0163** |  | **0.8982** |  | **0.9645** |
| **126** |  |  | **142** |  | **0** | **2** |  |  | **0.9396** |  | **0.0163** |  | **0.8982** |  | **0.9645** |
| **127** |  |  | **140** |  | **0** | **1** |  |  | **0.9396** |  | **0.0163** |  | **0.8982** |  | **0.9645** |
| **128** |  |  | **139** |  | **0** | **2** |  |  | **0.9396** |  | **0.0163** |  | **0.8982** |  | **0.9645** |
| **129** |  |  | **137** |  | **0** | **8** |  |  | **0.9396** |  | **0.0163** |  | **0.8982** |  | **0.9645** |
| **130** |  |  | **129** |  | **0** | **8** |  |  | **0.9396** |  | **0.0163** |  | **0.8982** |  | **0.9645** |
| **131** |  |  | **121** |  | **0** | **5** |  |  | **0.9396** |  | **0.0163** |  | **0.8982** |  | **0.9645** |
| **132** |  |  | **116** |  | **0** | **13** |  |  | **0.9396** |  | **0.0163** |  | **0.8982** |  | **0.9645** |
| **133** |  |  | **103** |  | **0** | **3** |  |  | **0.9396** |  | **0.0163** |  | **0.8982** |  | **0.9645** |
| **134** |  |  | **100** |  | **0** | **2** |  |  | **0.9396** |  | **0.0163** |  | **0.8982** |  | **0.9645** |
| **135** |  |  | **98** |  | **0** | **1** |  |  | **0.9396** |  | **0.0163** |  | **0.8982** |  | **0.9645** |
| **136** |  |  | **97** |  | **0** | **2** |  |  | **0.9396** |  | **0.0163** |  | **0.8982** |  | **0.9645** |
| **137** |  |  | **95** |  | **0** | **4** |  |  | **0.9396** |  | **0.0163** |  | **0.8982** |  | **0.9645** |
| **138** |  |  | **91** |  | **0** | **3** |  |  | **0.9396** |  | **0.0163** |  | **0.8982** |  | **0.9645** |
| **139** |  |  | **88** |  | **0** | **2** |  |  | **0.9396** |  | **0.0163** |  | **0.8982** |  | **0.9645** |
| **141** |  |  | **86** |  | **0** | **5** |  |  | **0.9396** |  | **0.0163** |  | **0.8982** |  | **0.9645** |
| **142** |  |  | **81** |  | **0** | **1** |  |  | **0.9396** |  | **0.0163** |  | **0.8982** |  | **0.9645** |

| **143** | **80** | **0** | **1** | **0.9396** |  | **0.0163** |  | **0.8982** |  | **0.9645** |
| --- | --- | --- | --- | --- | --- | --- | --- | --- | --- | --- |
| **144** | **79** | **0** | **1** | **0.9396** |  | **0.0163** |  | **0.8982** |  | **0.9645** |
| **145** | **78** | **0** | **2** | **0.9396** |  | **0.0163** |  | **0.8982** |  | **0.9645** |
| **146** | **76** | **1** | **0** | **0.9272** |  | **0.0202** |  | **0.8755** |  | **0.9579** |
| **147** | **75** | **0** | **2** | **0.9272** |  | **0.0202** |  | **0.8755** |  | **0.9579** |
| **149** | **73** | **0** | **3** | **0.9272** |  | **0.0202** |  | **0.8755** |  | **0.9579** |
| **150** | **70** | **0** | **1** | **0.9272** |  | **0.0202** |  | **0.8755** |  | **0.9579** |
| **151** | **69** | **0** | **3** | **0.9272** |  | **0.0202** |  | **0.8755** |  | **0.9579** |
| **152** | **66** | **0** | **4** | **0.9272** |  | **0.0202** |  | **0.8755** |  | **0.9579** |
| **153** | **62** | **0** | **2** | **0.9272** |  | **0.0202** |  | **0.8755** |  | **0.9579** |
| **154** | **60** | **0** | **4** | **0.9272** |  | **0.0202** |  | **0.8755** |  | **0.9579** |
| **155** | **56** | **0** | **1** | **0.9272** |  | **0.0202** |  | **0.8755** |  | **0.9579** |
| **156** | **55** | **0** | **1** | **0.9272** |  | **0.0202** |  | **0.8755** |  | **0.9579** |
| **157** | **54** | **0** | **4** | **0.9272** |  | **0.0202** |  | **0.8755** |  | **0.9579** |
| **158** | **50** | **0** | **2** | **0.9272** |  | **0.0202** |  | **0.8755** |  | **0.9579** |
| **159** | **48** | **0** | **3** | **0.9272** |  | **0.0202** |  | **0.8755** |  | **0.9579** |
| **162** | **45** | **0** | **1** | **0.9272** |  | **0.0202** |  | **0.8755** |  | **0.9579** |
| **163** | **44** | **0** | **2** | **0.9272** |  | **0.0202** |  | **0.8755** |  | **0.9579** |
| **164** | **42** | **0** | **1** | **0.9272** |  | **0.0202** |  | **0.8755** |  | **0.9579** |
| **165** | **41** | **0** | **1** | **0.9272** |  | **0.0202** |  | **0.8755** |  | **0.9579** |
| **166** | **40** | **0** | **1** | **0.9272** |  | **0.0202** |  | **0.8755** |  | **0.9579** |
| **167** | **39** | **0** | **1** | **0.9272** |  | **0.0202** |  | **0.8755** |  | **0.9579** |
| **169** | **38** | **0** | **2** | **0.9272** |  | **0.0202** |  | **0.8755** |  | **0.9579** |
| **170** | **36** | **0** | **3** | **0.9272** |  | **0.0202** |  | **0.8755** |  | **0.9579** |
| **171** | **33** | **0** | **2** | **0.9272** |  | **0.0202** |  | **0.8755** |  | **0.9579** |
| **174** | **31** | **0** | **3** | **0.9272** |  | **0.0202** |  | **0.8755** |  | **0.9579** |
| **177** | **28** | **0** | **1** | **0.9272** |  | **0.0202** |  | **0.8755** |  | **0.9579** |
| **178** | **27** | **0** | **1** | **0.9272** |  | **0.0202** |  | **0.8755** |  | **0.9579** |
| **179** | **26** | **0** | **1** | **0.9272** |  | **0.0202** |  | **0.8755** |  | **0.9579** |
| **181** | **25** | **1** | **0** | **0.8901** |  | **0.0412** |  | **0.7759** |  | **0.9480** |
| **185** | **24** | **0** | **2** | **0.8901** |  | **0.0412** |  | **0.7759** |  | **0.9480** |
| **187** | **22** | **0** | **1** | **0.8901** |  | **0.0412** |  | **0.7759** |  | **0.9480** |
| **200** | **21** | **0** | **1** | **0.8901** |  | **0.0412** |  | **0.7759** |  | **0.9480** |
| **203** | **20** | **0** | **2** | **0.8901** |  | **0.0412** |  | **0.7759** |  | **0.9480** |
| **208** | **18** | **0** | **1** | **0.8901** |  | **0.0412** |  | **0.7759** |  | **0.9480** |
| **229** | **17** | **0** | **2** | **0.8901** |  | **0.0412** |  | **0.7759** |  | **0.9480** |
| **233** | **15** | **0** | **1** | **0.8901** |  | **0.0412** |  | **0.7759** |  | **0.9480** |
| **234** | **14** | **0** | **3** | **0.8901** |  | **0.0412** |  | **0.7759** |  | **0.9480** |
| **237** | **11** | **0** | **1** | **0.8901** |  | **0.0412** |  | **0.7759** |  | **0.9480** |
| **238** | **10** | **0** | **2** | **0.8901** |  | **0.0412** |  | **0.7759** |  | **0.9480** |
| **239** | **8** | **0** | **2** | **0.8901** |  | **0.0412** |  | **0.7759** |  | **0.9480** |
| **240** | **6** | **0** | **1** | **0.8901** |  | **0.0412** |  | **0.7759** |  | **0.9480** |
| **241** | **5** | **0** | **1** | **0.8901** |  | **0.0412** |  | **0.7759** |  | **0.9480** |
| **242** | **4** | **0** | **3** | **0.8901** |  | **0.0412** |  | **0.7759** |  | **0.9480** |
| **246** | **1** | **0** | **1** | **0.8901** |  | **0.0412** |  | **0.7759** |  | **0.9480** |
|  |  |  |  |  |  |  |  |  |  |  |

**. stci, rmean dd(2)**

**failure _d: EstadFin == 1 analysis time _t: TSm**

|  | **Number of subjects** | **Restricted**  **mean** | **Std.** | **Err.** | **[95% Conf.** | **Interval]** |
| --- | --- | --- | --- | --- | --- | --- |
| **Total** | **221** | **230.31(*)** | **4.17** | | **222.15** | **238.48** |

**(*)**

**largest observed analysis time is censored, mean is underestimated**

## Completed Cumulative Survival Probabiliies. Life-Table or Acturial method. Entire Cohort

The cumulative survival probability of the Arpe prosthesis up to 10 years (120 months) was 93.96% and 88.84% up to 252 months.

**. ltable TSm _d, graph survival interval(6)**

|  | | | | | **Beg.** | | |  | | |  |  | | | | **Std.** |  | | | |
| --- | --- | --- | --- | --- | --- | --- | --- | --- | --- | --- | --- | --- | --- | --- | --- | --- | --- | --- | --- | --- |
| **Interval** | | | | | **Total** | | | **Deaths** | | | **Lost** | **Survival** | | | | **Error** | **[95% Conf. Int.]** | | | |
|  |  |  |  |  |  |  |  |  |  |  |  |  |  |  |  |  |  |  |  | |
| **0** |  |  | **6** |  |  | **221** |  |  | **1** | **1** |  |  | **0.9955** |  | **0.0045** |  | **0.9682** |  | **0.9994** | |
| **6** |  |  | **12** |  |  | **219** |  |  | **3** | **0** |  |  | **0.9818** |  | **0.0090** |  | **0.9523** |  | **0.9931** | |
| **12** |  |  | **18** |  |  | **216** |  |  | **2** | **2** |  |  | **0.9727** |  | **0.0110** |  | **0.9402** |  | **0.9876** | |
| **24** |  |  | **30** |  |  | **212** |  |  | **0** | **1** |  |  | **0.9727** |  | **0.0110** |  | **0.9402** |  | **0.9876** | |
| **48** |  |  | **54** |  |  | **211** |  |  | **0** | **1** |  |  | **0.9727** |  | **0.0110** |  | **0.9402** |  | **0.9876** | |
| **54** |  |  | **60** |  |  | **210** |  |  | **1** | **0** |  |  | **0.9681** |  | **0.0119** |  | **0.9342** |  | **0.9846** | |
| **60** |  |  | **66** |  |  | **209** |  |  | **0** | **4** |  |  | **0.9681** |  | **0.0119** |  | **0.9342** |  | **0.9846** | |
| **72** |  |  | **78** |  |  | **205** |  |  | **3** | **1** |  |  | **0.9539** |  | **0.0143** |  | **0.9159** |  | **0.9749** | |
| **78** |  |  | **84** |  |  | **201** |  |  | **1** | **0** |  |  | **0.9491** |  | **0.0150** |  | **0.9100** |  | **0.9715** | |
| **84** |  |  | **90** |  |  | **200** |  |  | **1** | **0** |  |  | **0.9444** |  | **0.0156** |  | **0.9041** |  | **0.9680** | |
| **90** |  |  | **96** |  |  | **199** |  |  | **0** | **1** |  |  | **0.9444** |  | **0.0156** |  | **0.9041** |  | **0.9680** | |
| **96** |  |  | **102** |  |  | **198** |  |  | **1** | **1** |  |  | **0.9396** |  | **0.0163** |  | **0.8982** |  | **0.9645** | |
| **108** |  |  | **114** |  |  | **196** |  |  | **0** | **1** |  |  | **0.9396** |  | **0.0163** |  | **0.8982** |  | **0.9645** | |
| **114** |  |  | **120** |  |  | **195** |  |  | **0** | **2** |  |  | **0.9396** |  | **0.0163** |  | **0.8982** |  | **0.9645** | |
| **120** |  |  | **126** |  |  | **193** |  |  | **0** | **51** |  |  | **0.9396** |  | **0.0163** |  | **0.8982** |  | **0.9645** | |
| **126** |  |  | **132** |  |  | **142** |  |  | **0** | **26** |  |  | **0.9396** |  | **0.0163** |  | **0.8982** |  | **0.9645** | |
| **132** |  |  | **138** |  |  | **116** |  |  | **0** | **25** |  |  | **0.9396** |  | **0.0163** |  | **0.8982** |  | **0.9645** | |
| **138** |  |  | **144** |  |  | **91** |  |  | **0** | **12** |  |  | **0.9396** |  | **0.0163** |  | **0.8982** |  | **0.9645** | |
| **144** |  |  | **150** |  |  | **79** |  |  | **1** | **8** |  |  | **0.9271** |  | **0.0203** |  | **0.8751** |  | **0.9579** | |
| **150** |  |  | **156** |  |  | **70** |  |  | **0** | **15** |  |  | **0.9271** |  | **0.0203** |  | **0.8751** |  | **0.9579** | |
| **156** |  |  | **162** |  |  | **55** |  |  | **0** | **10** |  |  | **0.9271** |  | **0.0203** |  | **0.8751** |  | **0.9579** | |
| **162** |  |  | **168** |  |  | **45** |  |  | **0** | **7** |  |  | **0.9271** |  | **0.0203** |  | **0.8751** |  | **0.9579** | |
| **168** |  |  | **174** |  |  | **38** |  |  | **0** | **7** |  |  | **0.9271** |  | **0.0203** |  | **0.8751** |  | **0.9579** | |
| **174** |  |  | **180** |  |  | **31** |  |  | **0** | **6** |  |  | **0.9271** |  | **0.0203** |  | **0.8751** |  | **0.9579** | |
| **180** |  |  | **186** |  |  | **25** |  |  | **1** | **2** |  |  | **0.8884** |  | **0.0425** |  | **0.7699** |  | **0.9479** | |
| **186** |  |  | **192** |  |  | **22** |  |  | **0** | **1** |  |  | **0.8884** |  | **0.0425** |  | **0.7699** |  | **0.9479** | |
| **198** |  |  | **204** |  |  | **21** |  |  | **0** | **3** |  |  | **0.8884** |  | **0.0425** |  | **0.7699** |  | **0.9479** | |
| **204** |  |  | **210** |  |  | **18** |  |  | **0** | **1** |  |  | **0.8884** |  | **0.0425** |  | **0.7699** |  | **0.9479** | |
| **228** |  |  | **234** |  |  | **17** |  |  | **0** | **3** |  |  | **0.8884** |  | **0.0425** |  | **0.7699** |  | **0.9479** | |
| **234** |  |  | **240** |  |  | **14** |  |  | **0** | **8** |  |  | **0.8884** |  | **0.0425** |  | **0.7699** |  | **0.9479** | |
| **240** |  |  | **246** |  |  | **6** |  |  | **0** | **5** |  |  | **0.8884** |  | **0.0425** |  | **0.7699** |  | **0.9479** | |
| **246** |  |  | **252** |  |  | **1** |  |  | **0** | **1** |  |  | **0.8884** |  | **0.0425** |  | **0.7699** |  | **0.9479** | |
|  |  |  |  |  |  |  |  |  |  |  |  |  |  |  |  |  |  |  |  | |

**.**

The cumulative survival probability of the Arpe prosthesis up to 10 years (120 months) was 93.96% and 88.84% up to 252 months.

**Comparison of survival function by Size of Trapezium**

### Log-rank test for equality of survivor functions

| **Trap_Size** | **Events Events**  **observed expected** |
| --- | --- |
| **Large** | **0 0.83** |
| **Medium** | **5 10.82** |
| **Small** | **10 3.35** |
| **Total** | **15 15.00** |

chi2(2) = 17.47

[Pr>chi2 = 0.0002](#_TOC_250001)

[Test for trend of survivor functions](#_TOC_250000)

chi2(1) = 15.31

### Pr>chi2 =

**0.0001**

### Tarone-Ware test for equality of survivor functions

| **Trap_Size** | **Events observed** | **Events expected** | **Sum of ranks** |
| --- | --- | --- | --- |
| **Large** | **0** | **0.83** | **-9.8089575** |
| **Medium** | **5** | **10.82** | **-82.136817** |
| **Small** | **10** | **3.35** | **91.945775** |
| **Total** | **15** | **15.00** | **0** |

**chi2(2) = 16.72**

### Pr>chi2 = 0.0002

### Test for trend of survivor functions

### chi2(1) = 14.95

**Pr>chi2 =**

**0.0001**

### Wilcoxon (Breslow) test for equality of survivor functions

| **Trap_Size** | **Events observed** | **Events expected** | **Sum of ranks** |
| --- | --- | --- | --- |
| **Large** | **0** | **0.83** | **-129** |
| **Medium** | **5** | **10.82** | **-1163** |
| **Small** | **10** | **3.35** | **1292** |
| **Total** | **15** | **15.00** | **0** |

### chi2(2) = 15.90

### Pr>chi2 = 0.0004

### Test for trend of survivor functions

### chi2(1) = 14.22

**Pr>chi2 =**

**0.0002**

There was a significant association between the survival curves of the Arpe prosthesis and the size of the trapezium, with a significant tendency for the survival curve to be higher as the trapezium is larger.

Taking into account the previous results, the small size of the trapezium was taken as an exposure factor for the failure of the prosthesis. A fact that was also verified with the same survival curve comparison tests (See below)

### Log-rank test for equality of survivor functions

| **SmallTrapez** | **Events Events**  **observed expected** |
| --- | --- |
| **No** | **5 11.65** |
| **Yes** | **10 3.35** |
| **Total** | **15 15.00** |

**chi2(1) = 17.24**

### Pr>chi2 =

**0.0000**

**. sts test SmallTrapez , tware**

### failure _d: EstadFin == 1 analysis time _t: TSm

**Tarone-Ware test for equality of survivor functions**

| **SmallTrapez** | **Events observed** | **Events expected** | **Sum of ranks** |
| --- | --- | --- | --- |
| **No** | **5** | **11.65** | **-91.945775** |
| **Yes** | **10** | **3.35** | **91.945775** |
| **Total** | **15** | **15.00** | **0** |

### chi2(1) = 16.55

**Pr>chi2 =**

**0.0000**

### . sts test SmallTrapez , wilcoxon

**failure _d: EstadFin == 1 analysis time _t: TSm**

### Wilcoxon (Breslow) test for equality of survivor functions

| **SmallTrapez** | **Events observed** | **Events expected** | **Sum of ranks** |
| --- | --- | --- | --- |
| **No** | **5** | **11.65** | **-1292** |
| **Yes** | **10** | **3.35** | **1292** |
| **Total** | **15** | **15.00** | **0** |

**chi2(1) = 15.77**

**Pr>chi2 =**

**0.0001**

# Analysis of confounders and interactions. Multivariate Cox Analysis

1. **Selection of potential confounder**

| **No. of subjects** | **=** | **221** | **Number of obs** | **=** | **221** |
| --- | --- | --- | --- | --- | --- |
| **No. of failures** | **=** | **15** |  |  |  |
| **Time at risk** | **=** | **30335** |  |  |  |
|  |  |  | **LR chi2(1)** | **=** | **0.03** |
| **Log likelihood** | **=** | **-76.994766** | **Prob > chi2** | **=** | **0.8609** |

| **_t** | **Haz. Ratio** | **Std. Err.** | **z** | **P>\|z\|** | **[95% Conf.** | **Interval]** |
| --- | --- | --- | --- | --- | --- | --- |
| **Age** | **.9937638** | **.0355131** | **-0.18** | **0.861** | **.926541** | **1.065864** |

| **No.** | **of subjects** | **=** | **221** | **Number of obs** | **=** | **221** |
| --- | --- | --- | --- | --- | --- | --- |
| **No.** | **of failures** | **=** | **15** |  |  |  |
| **Time at risk** | | **=** | **30335** |  |  |  |
|  | |  |  | **LR chi2(1)** | **=** | **1.43** |
| **Log likelihood** | | **=** | **-76.297213** | **Prob > chi2** | **=** | **0.2325** |

| **_t** | **Haz. Ratio** | **Std. Err.** | **z** | **P>\|z\|** | **[95%** | **Conf.** | **Interval]** |
| --- | --- | --- | --- | --- | --- | --- | --- |
| **Gender** | **5.83e-16** | **2.82e-08** | **-0.00** | **1.000** | **0** | | **.** |

| **No.** | **of subjects** | **=** | **221** | **Number of obs** | **=** | **221** |
| --- | --- | --- | --- | --- | --- | --- |
| **No.** | **of failures** | **=** | **15** |  |  |  |
| **Time at risk** | | **=** | **30335** |  |  |  |
|  | |  |  | **LR chi2(1)** | **=** | **19.47** |
| **Log likelihood** | | **=** | **-67.276223** | **Prob > chi2** | **=** | **0.0000** |

| **_t** | **Haz. Ratio** | **Std. Err.** | **z** | **P>\|z\|** | **[95% Conf.** | **Interval]** |
| --- | --- | --- | --- | --- | --- | --- |
| **Complications** | **2.259751** | **.3445793** | **5.35** | **0.000** | **1.675964** | **3.046888** |

**Cox regression -- no ties**

| **No.** | **of subjects** | **=** | **221** | **Number of obs** | **=** | **221** |
| --- | --- | --- | --- | --- | --- | --- |
| **No.** | **of failures** | **=** | **15** |  |  |  |
| **Time at risk** | | **=** | **30335** |  |  |  |
|  | |  |  | **LR chi2(1)** | **=** | **3.24** |
| **Log likelihood** | | **=** | **-75.388823** | **Prob > chi2** | **=** | **0.0717** |

| **_t** | **Haz. Ratio** | **Std. Err.** | **z** | **P>\|z\|** | **[95% Conf.** | **Interval]** |
| --- | --- | --- | --- | --- | --- | --- |
| **TrapzShape** | **2.263875** | **1.001895** | **1.85** | **0.065** | **.9509262** | **5.38962** |

**Cox regression -- no ties**

| **No.** | **of subjects** | **=** | **221** | **Number of obs** | **=** | **221** |
| --- | --- | --- | --- | --- | --- | --- |
| **No.** | **of failures** | **=** | **15** |  |  |  |
| **Time at risk** | | **=** | **30335** |  |  |  |
|  | |  |  | **LR chi2(1)** | **=** | **3.31** |
| **Log likelihood** | | **=** | **-75.354183** | **Prob > chi2** | **=** | **0.0688** |

| **_t** | **Haz. Ratio** | **Std. Err.** | **z** | **P>\|z\|** | **[95%** | **Conf.** | **Interval]** |
| --- | --- | --- | --- | --- | --- | --- | --- |
| **GradeIV_Eaton** | **5.26e-16** | **1.73e-08** | **-0.00** | **1.000** | **0** | | **.** |

# MMax. Maximum Model

**. stcox SmallTrapez Age Gender TrapzShape Complicat c.SmallTrapez#c.Age c.SmallTrapez#c.Gender**

**> c.SmallTrapez#i.TrapzShape c.SmallTrapez#i.Complicat**

**Cox regression -- no ties**

| **No. of subjects =** | **221** | **Number of obs** | **=** | **221** |
| --- | --- | --- | --- | --- |
| **No. of failures =** | **15** |  |  |  |
| **Time at risk =** | **30335** |  |  |  |
|  |  | **LR chi2(7)** | **=** | **92.83** |
| **Log likelihood =** | **-30.596999** | **Prob > chi2** | **=** | **0.0000** |

| **_t** | **Haz. Ratio** | **Std. Err.** | **Z** | **P>\|z\|** | **[95% Conf.** | **Interval]** |
| --- | --- | --- | --- | --- | --- | --- |
| **SmallTrapez** | **1.695464** | **11.13882** | **0.08** | **0.936** | **4.34e-06** | **662969.2** |
| **Age** | **.8810936** | **.0959274** | **-1.16** | **0.245** | **.7117854** | **1.090674** |
| **Gender** | **.00002** | **.** | **.** | **.** | **.** | **.** |
| **TrapzShape** | **2.556319** | **1.527432** | **1.57** | **0.116** | **.7925223** | **8.245529** |
| **Complicat** | **4.90e+17** | **2.08e+25** | **0.00** | **1.000** | **0** | **.** |
| **c.SmallTrapez#c.Age** | **1.010531** | **.122039** | **0.09** | **0.931** | **.7975396** | **1.280403** |
| **c.SmallTrapez#c.Gender** | **.0000132** | **.** | **.** | **.** | **.** | **.** |
| **TrapzShape#c.SmallTrapez** |  |  |  |  |  |  |
| **Displacic** | **.3940014** | **.3717579** | **-0.99** | **0.324** | **.0619942** | **2.504058** |
| **Great CMC subluxation** | **.0093162** | **.0181933** | **-2.39** | **0.017** | **.0002027** | **.42808** |
| **Complicat#c.SmallTrapez** |  |  |  |  |  |  |
| **Yes** | **8.879345** | **.** | **.** | **.** | **.** | **.** |

1. **MMaxNoInteract. Maximum model without the interactions between the exposure (Small_Trapz) and the cofounders**

| **No. of subjects** | **=** | **221** | **Number of obs** | **=** | **221** |
| --- | --- | --- | --- | --- | --- |
| **No. of failures** | **=** | **15** |  |  |  |
| **Time at risk** | **=** | **30335** |  |  |  |
|  |  |  | **LR chi2(3)** | **=** | **84.67** |
| **Log likelihood** | **=** | **-34.673448** | **Prob > chi2** | **=** | **0.0000** |

| **_t** | **Haz. Ratio** | **Std. Err.** | **z** | **P>\|z\|** | **[95% Conf.** | **Interval]** |
| --- | --- | --- | --- | --- | --- | --- |
| **SmallTrapez** | **3.639171** | **2.477723** | **1.90** | **0.058** | **.9582161** | **13.82107** |
| **Age** | **.9520679** | **.0369557** | **-1.27** | **0.206** | **.8823228** | **1.027326** |
| **Gender** | **.000941** | **.** | **.** | **.** | **.** | **.** |
| **TrapzShape** | **.8220775** | **.3280789** | **-0.49** | **0.623** | **.37602** | **1.797275** |
| **Complicat** | **5.91e+17** | **.** | **.** | **.** | **.** | **.** |

# Analysis of interactions. Global Chunk Test

| **. lrtest MMax MMaxNoInterac**  **Likelihood-ratio test** | **LR chi2(4)** | **=** | **8.15** |
| --- | --- | --- | --- |
| **(Assumption: MMaxNoInterac nested in MMax)** | **Prob > chi2** | **=** | **0.0861** |

Since the Global Test was not significant (p= 0.0861), all interactions can be excluded from the final “Reference model”.

# Confounding Análisis

**CONFOUND - Cox regression**

**ALL VARIABLES**

**Potential confounders: Age Gender TrapzShape Complicat**

**Exposition: SmallTrapez**

**Important change: >= 10%**

**Variable**

**Valid**

**Missing**

**SmallTrapez**

**Age Gender TrapzShape Complicat**

**221**

**221**

**221**

**221**

**221**

**0**

**0**

**0**

**0**

**0**

**Valid number of cases (listwise): 221**

**A new dataset has been created with the results. Save your data and execute: use "confound_results.dta", clear to open the dataset with the results**

**. list, clean noobs**

| **NVar**  **5** | **Variables**  **SmallTrapez** | **Age Gender TrapzShape Complicat** | **ExpB**  **3.639171** | **Change**  **0** | **lbCI**  **.9582161** |  | **ubCI**  **13.82107** |  | **Range**  **12.86285** | **RangeDiff**  **0** | **Select**  **.** |
| --- | --- | --- | --- | --- | --- | --- | --- | --- | --- | --- | --- |
| **4** | **SmallTrapez** | **Age TrapzShape Complicat** | **3.639171** | **0** | **.9582161** |  | **13.82107** |  | **12.86285** | **0** | **1** |
| **3** | **SmallTrapez** | **TrapzShape Complicat** | **2.452399** | **32.61106** | **.7585421** |  | **7.92871** |  | **7.170168** | **-5.692681** | **0** |
| **4** | **SmallTrapez** | **Gender TrapzShape Complicat** | **2.452399** | **32.61106** | **.7585421** |  | **7.92871** |  | **7.170168** | **-5.692681** | **0** |
| **2** | **SmallTrapez** | **Complicat** | **2.480053** | **31.85117** | **.7701056** |  | **7.986777** |  | **7.216671** | **-5.646178** | **0** |
| **3** | **SmallTrapez** | **Gender Complicat** | **2.480053** | **31.85117** | **.7701056** |  | **7.986777** |  | **7.216671** | **-5.646178** | **0** |
| **4** | **SmallTrapez** | **Age Gender Complicat** | **3.218** | **11.57327** | **.9448029** |  | **10.96052** |  | **10.01571** | **-2.847136** | **0** |
| **3** | **SmallTrapez** | **Age Complicat** | **3.218** | **11.57327** | **.9448029** |  | **10.96052** |  | **10.01571** | **-2.847136** | **0** |
| **3** | **SmallTrapez** | **Gender TrapzShape** | **7.056549** | **93.90536** | **2.317324** |  | **21.4881** |  | **19.17078** | **6.307927** | **0** |
| **2** | **SmallTrapez** | **Gender** | **7.256579** | **99.40195** | **2.430392** |  | **21.66643** |  | **19.23604** | **6.373193** | **0** |
| **4** | **SmallTrapez** | **Age Gender TrapzShape** | **7.11331** | **95.4651** | **2.318754** |  | **21.82171** |  | **19.50296** | **6.640111** | **0** |
| **2** | **SmallTrapez** | **TrapzShape** | **7.163243** | **96.8372** | **2.344415** |  | **21.88693** |  | **19.54252** | **6.679669** | **0** |
| **1** | **SmallTrapez** |  | **7.373491** | **102.6146** | **2.463824** |  | **22.06667** |  | **19.60284** | **6.739993** | **0** |
| **3** | **SmallTrapez** | **Age TrapzShape** | **7.177759** | **97.23608** | **2.341356** |  | **22.00444** |  | **19.66308** | **6.800231** | **0** |
| **2** | **SmallTrapez** | **Age** | **7.610003** | **109.1136** | **2.516934** |  | **23.009** |  | **20.49207** | **7.62922** | **0** |
| **3** | **SmallTrapez** | **Age Gender** | **7.599536** | **108.826** | **2.509545** |  | **23.01331** |  | **20.50377** | **7.64092** | **0** |

Comparison of reduced models with the reference model. ExpB = Exponential Regression coefficient = HR (Hazard Ratio); Change = change in the effect with the reference model including all confounders. Selection of best models: “Select = 1”, those models with a change in effect less than 10% compared with the reference model. Of those models with Select=1, models with a narrower 95% CI (lower range) should be selected, coincidental with a range difference with a more negative value compared with the full model. In case of similar range difference, the more parsimonious model should be selected. Observe that the best model selected was “Small Trapezium” as exposure variable, and Age, Trapezium Shape and Complications as confounders.

15

**Cox regression -- no ties**

| **No. of subjects =** | **221** | **Number of obs** | **=** | **221** |
| --- | --- | --- | --- | --- |
| **No. of failures =** | **15** |  |  |  |
| **Time at risk =** | **30335** |  |  |  |
|  |  | **LR chi2(1)** | **=** | **13.75** |
| **Log likelihood =** | **-70.13677** | **Prob > chi2** | **=** | **0.0002** |

| **_t** | **Haz. Ratio** | **Std. Err.** | **z** | **P>\|z\|** | **[95% Conf.** | **Interval]** |
| --- | --- | --- | --- | --- | --- | --- |
| **SmallTrapez** | **7.373491** | **4.123877** | **3.57** | **0.000** | **2.463824** | **22.06667** |

If the confounding variables were not taken into account, as shown above (Unadjusted model), there was a significant association between the small trapezium and the risk rate of prosthesis failure, such that it multiplied the HR of failure by 7.37 being significant.

When adjusting for confounding variables, the effect decreases the HR to 3.64 being not significant. The multivariate analysis showed that the best model to explain the association between the risk rate of prosthesis failure and small trapezium was the model adjusted for age, trapezius shape, and the existence of complications. When the results were adjusted for the confounding variables.

**Cox regression -- no ties**

| **No.** | **of subjects** | **=** | **221** | **Number of obs** | **=** | **221** |
| --- | --- | --- | --- | --- | --- | --- |
| **No.** | **of failures** | **=** | **15** |  |  |  |
| **Time at risk** | | **=** | **30335** |  |  |  |
|  | |  |  | **LR chi2(4)** | **=** | **84.67** |
| **Log likelihood** | | **=** | **-34.673448** | **Prob > chi2** | **=** | **0.0000** |

| **_t** | **Haz. Ratio** | **Std. Err.** | **z** | **P>\|z\|** | **[95% Conf.** | **Interval]** |
| --- | --- | --- | --- | --- | --- | --- |
| **SmallTrapez** | **3.639171** | **2.477723** | **1.90** | **0.058** | **.9582161** | **13.82107** |
| **Age** | **.9520679** | **.0369557** | **-1.27** | **0.206** | **.8823228** | **1.027326** |
| **TrapzShape** | **.8220775** | **.3280789** | **-0.49** | **0.623** | **.37602** | **1.797275** |
| **Complicat** | **7.99e+16** | **1.52e+24** | **0.00** | **1.000** | **0** | **.** |

## Diagnosis of the Best Cox Model

**Cox regression -- no ties**

| **No.** | **of subjects** | **=** | **221** | **Number of obs** | **=** | **221** |
| --- | --- | --- | --- | --- | --- | --- |
| **No.** | **of failures** | **=** | **15** |  |  |  |
| **Time at risk** | | **=** | **30335** |  |  |  |
|  | |  |  | **LR chi2(4)** | **=** | **84.67** |
| **Log likelihood** | | **=** | **-34.673448** | **Prob > chi2** | **=** | **0.0000** |

| **_t** | **Haz. Ratio** | **Std. Err.** | **z** | **P>\|z\|** | **[95% Conf.** | **Interval]** |
| --- | --- | --- | --- | --- | --- | --- |
| **SmallTrapez** | **3.639171** | **2.477723** | **1.90** | **0.058** | **.9582161** | **13.82107** |
| **Age** | **.9520679** | **.0369557** | **-1.27** | **0.206** | **.8823228** | **1.027326** |
| **TrapzShape** | **.8220775** | **.3280789** | **-0.49** | **0.623** | **.37602** | **1.797275** |
| **Complicat** | **7.99e+16** | **1.52e+24** | **0.00** | **1.000** | **0** | **.** |

1. The null hypothesis (H0) assumed that the coefficients of interaction between predictors (SmallTrapez Age TrapzShape Complicat) with the survival time variable will be statistically equal to zero.

Proportionality assumption.

**Cox regression -- no ties**

| **No. of subjects =** | **221** | **Number of obs** | **=** | **221** |
| --- | --- | --- | --- | --- |
| **No. of failures =** | **15** |  |  |  |
| **Time at risk =** | **30335** |  |  |  |
|  |  | **LR chi2(6)** | **=** | **85.39** |
| **Log likelihood =** | **-34.315099** | **Prob > chi2** | **=** | **0.0000** |

| **_t** | **Haz. Ratio** | **Std. Err.** | **z** | **P>\|z\|** | **[95% Conf.** | **Interval]** |
| --- | --- | --- | --- | --- | --- | --- |
| **main SmallTrapez**  **Age TrapzShape Complicat** | **2.47315**  **.932746**  **.822436 1.33e+17** | **2.592152**  **.0633378**  **.5411006**  **.** | **0.86**  **-1.03**  **-0.30**  **.** | **0.388**  **0.305**  **0.766**  **.** | **.3170231**  **.8165124**  **.2265041**  **.** | **19.29345**  **1.065526**  **2.986264**  **.** |
| **tvc**  **SmallTrapez**  **Age TrapzShape Complicat** | **1.007568**  **1.000411**  **.999595**  **1.110339** | **.0167994**  **.0011486**  **.0114389**  **.** | **0.45**  **0.36**  **-0.04**  **.** | **0.651**  **0.720**  **0.972**  **.** | **.9751735**  **.9981624**  **.9774247**  **.** | **1.041038**  **1.002665**  **1.022268**  **.** |

**Note: Variables in tvc equation interacted with _t.**

**. test ([tvc]: SmallTrapez Age TrapzShape Complicat)**

**( 1) [tvc]SmallTrapez = 0**

**( 2) [tvc]Age = 0**

**( 3) [tvc]TrapzShape = 0**

**( 4) [tvc]Complicat = 0**

**Constraint 4 dropped**

**chi2( 3) = 0.70**

**Prob > chi2 =**

**0.8743**

The global Chunk test demonstrated that the p = 0.8743 , not significant , consequently we retain the Ho , meant that we can accept the proportionality assumption.

Other way to analyze the proportionality assumption is by assessing the relationship between the Schoenfeld residuals and survival time which should be analyzed using a chi-square test, assuming proportionality when p is > 0.05.

**. estat phtest, detail**

**Test of proportional-hazards assumption**

**Time: Time**

| **0.6477** |
| --- |
| **0.8407** |
| **0.9306** |
| **1.0000** |

|  | **rho chi2 df Prob>chi2** |
| --- | --- |
| **SmallTrapez Age TrapzShape Complicat** | **0.10948 0.21 1**  **0.06042 0.04 1**  **-0.02640 0.01 1**  **. 0.00 1** |
| **global test** | **0.44 4 0.9788** |

The relationship between the Schoenfeld residuals and survival time analysis demonstrated the p values for each predictor (p=0.6477, p=0.8407, p= 0.9306, p= 1) and for the global test for all predictors together were not significant (p=0.9788), consequently there were not a relationship between the Schoenfeld residual and the survival time and the proportionality assumption was accepted.

1. **The log-linear relationship assumption** of the Cox model is that one in which it is assumed that the relationship between the instantaneous incidence rate of prosthesis failure and the explanatory variables must be log-linear. For this, the analysis of the squared linear predictor will be used and in this way, confirming that the squared predictor coefficient is not significant (p > 0.05).

B-1. For that purpose , the first way for analysing the log lineal relationship was by the assessment of the squared predictor coefficient ( pl = predictor coefficient)

**Cox regression -- no ties**

| **No.** | **of subjects** | **=** | **221** | **Number of obs** | **=** | **221** |
| --- | --- | --- | --- | --- | --- | --- |
| **No.** | **of failures** | **=** | **15** |  |  |  |
| **Time at risk** | | **=** | **30335** |  |  |  |
|  | |  |  | **LR chi2(2)** | **=** | **84.73** |
| **Log likelihood** | | **=** | **-34.645759** | **Prob > chi2** | **=** | **0.0000** |

| **_t** | **Coef.** | **Std. Err.** | **z** | **P>\|z\|** | **[95% Conf.** | **Interval]** |
| --- | --- | --- | --- | --- | --- | --- |
| **pl** | **15.80948** | **63.30667** | **0.25** | **0.803** | **-108.2693** | **139.8883** |
| **c.pl#c.pl** | **-.2012729** | **.8603235** | **-0.23** | **0.815** | **-1.887476** | **1.48493** |

Observe the p value of the squared pl coefficient (c.pl#c.pl) which was 0.815, not significant meant that the log lineal assumption was accepted.

B-2. Similar results using the linktest below.

**Cox regression -- no ties**

| **No.** | **of subjects** | **=** | **221** | **Number of obs** | **=** | **221** |
| --- | --- | --- | --- | --- | --- | --- |
| **No.** | **of failures** | **=** | **15** |  |  |  |
| **Time at risk** | | **=** | **30335** |  |  |  |
|  | |  |  | **LR chi2(2)** | **=** | **84.73** |
| **Log likelihood** | | **=** | **-34.645761** | **Prob > chi2** | **=** | **0.0000** |

| **_t** | **Coef.** | **Std. Err.** | **z** | **P>\|z\|** | **[95% Conf.** | **Interval]** |
| --- | --- | --- | --- | --- | --- | --- |
| **_hat** | **15.80879** | **63.30601** | **0.25** | **0.803** | **-108.2687** | **139.8863** |
| **_hatsq** | **-.2012634** | **.8603145** | **-0.23** | **0.815** | **-1.887449** | **1.484922** |

The diagnosis of the models demonstrated that the proportional hazard assumption was accepted based on Schoenfeld residuals (p = 0.9788) and by the test of interaction between survival time and the independent variables (p = 0.8743). The log linear assumption was also accepted (p = 0.815)
